# An investigation on fatigue, fracture resistance, and color properties of aesthetic CAD/CAM monolithic ceramics

**DOI:** 10.1007/s00784-022-04833-y

**Published:** 2022-12-27

**Authors:** Ahmed Mahmoud Fouda, Osama Atta, Mutlu Özcan, Bogna Stawarczyk, Robert Glaum, Christoph Bourauel

**Affiliations:** 1 Department of Oral Technology, University Hospital Bonn, Bonn University, Welschnonnenstr. 17, 53111 Bonn, Germany; 2 Department of Fixed Prosthodontics, Suez Canal University, Ismailia, Egypt; 3 Division of Dental Biomaterials, Center for Dental Medicine, Clinic for Reconstructive Dentistry, University of Zurich, Zurich, Switzerland; 4 Department of Prosthetic Dentistry, University Hospital, LMU Munich, Munich, Germany; 5 Institute of Inorganic Chemistry, Bonn University, Bonn, Germany

**Keywords:** Monolithic, Aging, Dental ceramics, Dental materials, CAD/CAM, Translucency, Color, Fracture resistance, Prosthodontics, Translucent zirconia, Lithium disilicate

## Abstract

**Objectives:**

To evaluate and compare fracture resistance, translucency, and color reproducibility, as well as the effect of aging on the fracture load and color stability of novel monolithic CAD/CAM ceramics.

**Materials and methods:**

One hundred crowns of uniform thickness were milled from five ceramic blocks (*n* = 20): partially crystallized lithium disilicate (PLD) and fully crystallized lithium disilicate (FLD), lithium metasilicate (LMS), 4Y-TZP (SMZ), and 5Y-TZP (UMZ) monolithic zirconia. PLD crowns were glazed, LMS was fired, and FLD was polished. SMZ and UMZ crowns were sintered and polished. Crowns were adhesively cemented to epoxy dies. Half of the crowns (*n* = 10) were subjected to 1.200.000 load cycles with thermal cycling. Color space values L, a, b defined by the Commission Internationale de l´Eclairage (CIELAB) were measured before and after aging, and (∆*E*) was calculated. Both aged and non-aged specimens were loaded until fracture in a universal testing machine and the fracture load was recorded. X-ray diffraction (XRD) and scanning electron microscope (SEM) fractographic analysis were carried out on fractured fragments of representative samples. For translucency and color reproducibility, 50 rectangular-shaped specimens were fabricated and processed as described previously. Color values were measured over black and white backgrounds, and the translucency parameter (TP) was computed. Using the shade verification mode, (∆*E*) to shade A3 was calculated. Data were statistically analyzed using one-way and two-way ANOVA, and *t*-test.

**Results:**

Aging did not affect fracture resistance significantly (*p* > 0.05). The highest mean fracture load was obtained for the SMZ and UMZ. A significant color change was observed after aging in all groups. The highest TP was noted for FLD. SMZ and UMZ had the best shade match.

**Conclusions:**

Zirconia showed higher fracture resistance and color stability than lithium silicate ceramics. Lithium silicate ceramics were more translucent. The experimental FLD demonstrated high translucency.

**Clinical relevance:**

Tested ceramics showed sufficient stability to withstand masticatory forces. Characterization of final restorations might be mandatory for better color match.

**Supplementary Information:**

The online version contains supplementary material available at 10.1007/s00784-022-04833-y.

## Introduction

In recent years, all-ceramic restorations have gained increased popularity owing to their excellent esthetics, biocompatibility, and ability to reproduce a natural-looking appearance [1]. Dental restorations are subjected to stresses of different types and magnitudes during mastication [2, 3]. The brittleness of ceramics makes them vulnerable to failure under stress [4]. Monolithic ceramics do not suffer from veneer problems such as chipping, delamination, and fracture, but other clinical concerns may exist, such as the ability to withstand masticatory loads, the attrition of antagonist teeth, and the ability to achieve satisfactory esthetics [5].

A variety of ceramic materials are available for CAD/CAM monolithic crown fabrication including silicate-based ceramics, high-strength polycrystalline ceramics, and polymer-infiltrated ceramic [6]. Silicate glass–ceramics are known for their high translucency, reduced elastic modulus, and the ability to be acid etched by hydrofluoric acid and adhesively cemented to underlying tooth [7, 8]. However, the initially introduced silicate glass–ceramics had low mechanical stability, which limited their use to anterior single-unit FDPs [9]. Various types of reinforcing phases have been added to the glass matrix over the years aiming to improve the fracture resistance of silicate glass–ceramics. Among these are leucite, lithium disilicate, lithium metasilicate, and lithium aluminasilicate [10].

Lithium disilicate (LD) glass–ceramic is commonly used in dental clinics due to its good mechanical properties, biocompatibility, and superior esthetic outcomes [11]. LD CAD/CAM blocks are supplied in a “soft” intermediate phase to facilitate the machining process and preserve edge stability, then the milled restoration must be fired in a special ceramic oven to achieve the ultimate flexural strength [12, 13]. Fully crystallized lithium disilicate (FLD) restorations were recently developed that can be milled directly from fully crystallized blocks without further crystallization [14]. However, it is not yet known how such a material will perform mechanically and aesthetically.

High-strength polycrystalline dental ceramics, such as yttrium stabilized tetragonal zirconia polycrystal (Y-TZP), have increased the use of metal-free restorations in dentistry because of their high flexural strength and fracture toughness [15]. The first and second generations of zirconia suffered from increased opacity and poor aesthetic performance [16]. Highly translucent zirconia (3rd and 4th generation) was recently developed and indicated for esthetic anterior restorations. The translucency was improved by increasing the yttria content to 4 mol% or 5 mol% to produce partially stabilized zirconia (PSZ), with a higher amount of cubic phase [7, 17]. The isotropic nature of the cubic crystals allows more even light transmittance in all spatial directions. In addition, the larger grain size (~ 1.5 µm) and higher volume of the cubic crystals allow more light transmittance at the grain boundaries [17]. However, the increase in translucency is associated with a marked decrease in flexural strength and fracture toughness [18].

A restoration is considered successful if it is able to restore function and aesthetics and to survive in the harsh oral environment for several years without fracture or changing color. The repetitive subcritical masticatory loads tend to initiate cracks inside the restoration from the voids and flaws originated during manufacturing or processing [19]. This so-called slow or subcritical crack growth progresses slowly over time and is accelerated in the presence of moisture [4]. The size and rate of crack growth strongly affect the fracture load of the restoration [3, 20, 21]. Therefore, fatigue experiments under thermal and cyclic loading, simulating the mastication, are more reliable predictors of the long-term mechanical performance of ceramic materials used in the oral environment than static load testing [22, 23].

Many CAD/CAM monolithic ceramics are available in form of pre-shaded blocks. Choosing the appropriate shade remains a challenge for dentists due to the lack of standardization in color conception between manufacturers and the available shade guide systems [24]. Additionally, the color stability over time is another concern. The effect of aging on the color of ceramic restorations has been reported in previous studies [25–29]. The aim of this study was to evaluate the fracture load and optical parameters of five monolithic CAD/CAM ceramic materials after thermomechanical aging. Further aims were to evaluate the translucency and color reproducibility of the tested materials. The null hypotheses of this study were:

1. The maximum fracture load will not differ among the tested materials
2. Thermomechanical aging will not affect the fracture load.
3. Thermomechanical aging will not affect the color of the tested crowns.
4. Different materials will not differ in ∆*E* values from the selected shade.

## Materials and methods

The materials used in this study, the manufacturers, and the groups are listed in Table 1. A schematic diagram showing the testing design is presented in Online Resource 1.

**Table 1:** Materials used in the study

Material	Group name	Product name	Manufacturer	Composition	Lot. Nr
Partially crystallized lithium disilicate	PLD	IPS e.max CAD	Ivoclar Vivadent, Schaan, Liechtenstein	57–80% SiO2; 11–19% Li2O; 0–13% K2O; 0–11% P2O5; 0–8% ZrO2; 0–8% ZnO; 0 = 5% Al2O3; 0–5% MgO; 0–8% coloring oxides	X25830
Experimental fully crystallized lithium disilicate	FLD	GC Initial LiSi Block	GC Corporation, Tokyo, Japan	No data available	1,904,251
Lithium metasilicate	LMS	Celtra Duo	Sirona Dentsply, Milford, DE, USA	58% SiO2; 10.1% ZrO2; 18.5% Li2O; others: 13.4	16,004,977
Super-translucent monolithic zirconia (4Y-TZP)	SMZ	Katana STML	Kuraray, Tokyo, Japan	87–92% ZrO2 + HfO2; 7–9% Y2O2; 0–2% other oxides	DZMFB
Ultra-translucent monolithic zirconia (5Y-TZP)	UMZ	Katana UTML		87–92% ZrO2 + HfO2; 8–11% Y2O2; 0–2% other oxides	DYWYA

### Fracture load

#### Specimens’ preparation

A prefabricated die for a prepared premolar with a heavy chamfer finish line (90 degrees cavosurface angle with a large radius rounded internal angle) was used in this study. For duplication, an impression was taken of the die with addition silicon impression material (Honigum putty and light, DMG Chemisch-Pharmazeutische Fabrik GmbH, Hamburg, Germany), checked with 2.5 × magnifying loupes under LED light for any defects, and poured with epoxy resin (Technovit® Epox, Kulzer GmbH, Hanau, Germany). The epoxy was allowed to set for 24 h and then examined under magnification for any defects or entrapped air bubbles. Defective dies were discarded and replaced.

The master die was scanned with Omnicam intraoral scanner (Dentsply Sirona; Bensheim, Germany). As the aim of our study was to measure the fracture load of anatomically shaped crowns, a monolithic crown restoration with anatomical cusps was designed using CEREC software v.4.6.1 (Dentsply Sirona; Bensheim, Germany). The occlusal surface was adjusted to 1.5 mm uniform thickness in the central groove and 1.5 mm axial wall thickness at the buccal and lingual contours. The wall thicknesses were ensured by using Cerec software tools “cursor details” and “show minimum thickness.” To ensure standardization of occlusal area among samples, a total of 100 monolithic crowns (*n* = 20/group) were milled from the same design using the 5-axis milling unit ceramill® motion 2 (Amann Girrbach AG, Koblach, Austria). All thicknesses were double checked manually after milling using a digital micrometer. All crowns were shape congruent. The sample size was decided based on the commonly used sample size for similar tests in the literature [27, 30–32].

Crowns of the PLD group were subjected to a combined glazing/crystallization cycle in a ceramic furnace (Programat P500 Oven, Ivoclar Vivadent AG, Schaan, Liechtenstein), while crowns of the LMS group were only subjected to a firing cycle without glazing. The firing of the restorations was done according to the protocols provided by the manufacturers (Table 2). FLD crowns were polished according to the manufacturer’s instructions using ceramic polishing kit (Panther GC edition, SUN GmbH, Ölbronn-Dürrn Germany). SMZ and UMZ crowns were sintered in a sintering furnace (Cercon Heat Plus; DeguDent GmbH, Hanau, Germany) and polished according to the manufacturer’s instructions.

**Table 2:** Firing parameters of PLD and ZLS specimens

Group	Standby temp	Closing time	1^st^ heating rate	Intermediate temp	Holding time	2^nd^ heating rate	Final temp	Holding time	Vacuum
PLD	403 °C	6 min	90 °C/min	820 °C	10 s	30 °C/min	840 °C	7 min	on
ZLS	500 °C	1 min	60 °C/min	-	-	-	820 °C	1 min	off

All crowns were cleaned in an ultrasonic cleaner (Bandelin electronic KG, Berlin. Germany), air-dried, and prepared for adhesive cementation. FLD, LMS, and FLD crowns were etched using 9% hydrofluoric acid (Porcelain Etch, Ultradent, South Jordan, USA) for 20 s, rinsed with water for 1 min, then air-dried. SMZ and UMZ crowns were air-abraded using alumina powder size 50 µm at 2 bar pressure for 10 s. Panavia V5 (Kuraray Europe GmbH, Hattersheim am Main, Germany) was used to cement the crowns to the epoxy dies. The ceramic primer (Clearfil Ceramic Primer Plus, Kuraray Europe GmbH, Hattersheim am Main, Germany) was applied to the fitting surface of the crown and left 1 min to dry. Meanwhile, in order to replicate all clinical steps during bonding, the tooth primer was applied to the epoxy die, left for 20 s, then thoroughly dried with mild air. Afterward, the cement was applied to the fitting surface of the crown and then the crown was seated slowly over the die with finger pressure, then a 3 kg static load was applied till the full set of the cement. The margins were photo-polymerized for 2–3 s, and the excess cement was removed; after that, every margin on each surface was photo-polymerized for 20 s.

#### Cyclic loading and fracture load testing

Half of the crowns (*n* = 10) were subjected to thermomechanical loading in a specially designed chewing simulator (Division of Dental Biomaterials, Zurich University, Switzerland) following a previously published protocol [33]. The crowns were subjected to 1.2 million load cycles at a force of 49 N and frequency of 1.7 Hz. The thermal cycling was done between 5 and 55 °C with a 60 s dwell time and 10 s for the water change. The load was applied to the central groove in a vertical direction perpendicular to the occlusal table. At the end of the cycles, the survived crowns were examined with a stereomicroscope at 10 × magnification to exclude any specimen that showed signs of fracture from further testing.

**Fig. 1:**
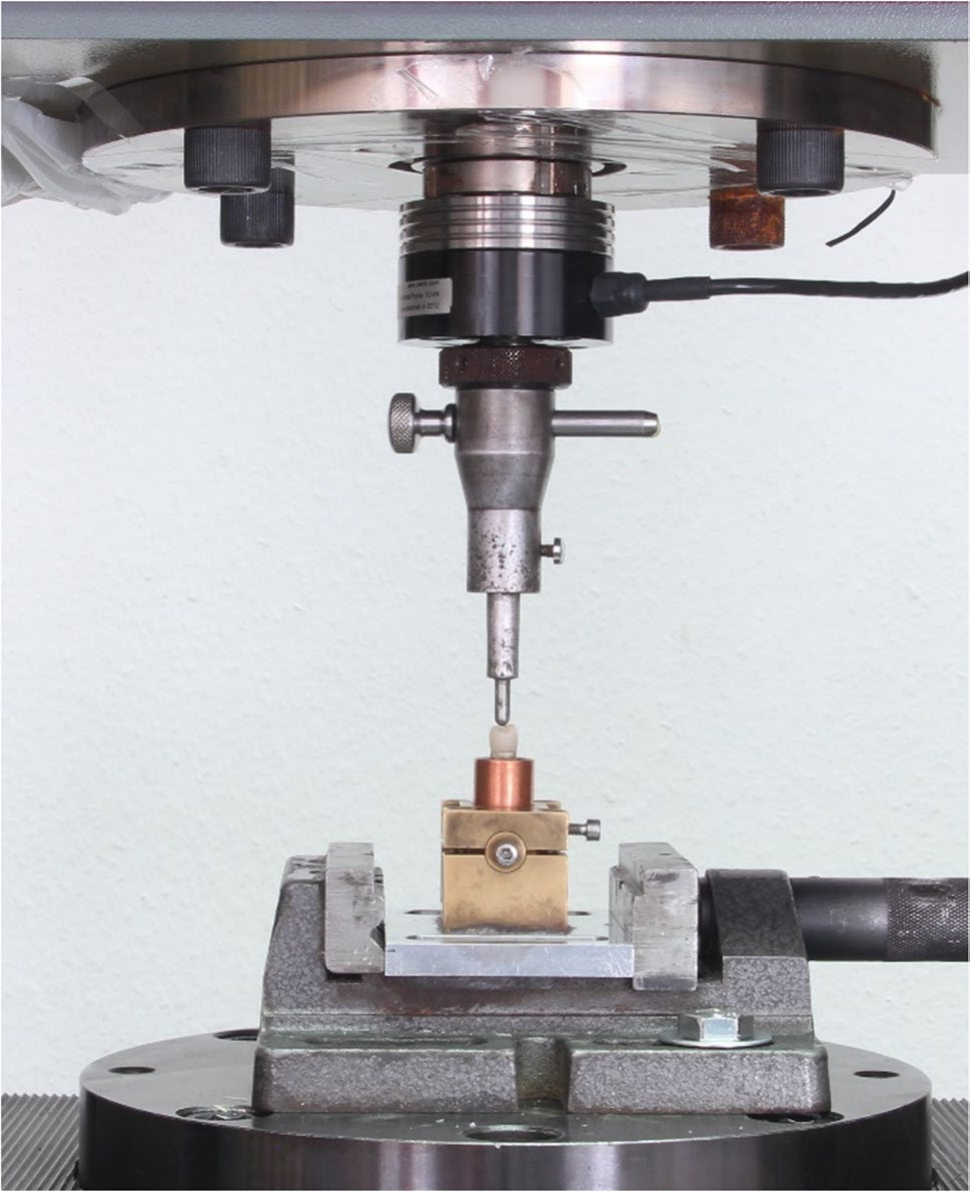
Load to fracture test using universal testing machine. The piston applied perpendicular to the occlusal surface and driven at a crosshead speed of 1 mm/min

The surviving specimens from the thermomechanical loading as well as the unfatigued specimens were loaded until fracture in a universal testing machine (Zwick ZmartPro, ZwickRoell GmbH & Co. KG, Ulm, Germany). The epoxy dies and the adhesively seated crowns were prepared for the fracture loading test. The base of the die was trimmed to fit centrally in a cylindrical copper tube (ø18 mm and L 5 mm) and fixed with auto-polymerized resin (Technovit 4004; Kulzer GmbH, Hanau, Germany). Afterward, the copper cylinder was fitted in a specially designed holder of the same diameter and tightly fixed in position using 3 screws. The holder was securely attached to the Zwick table with the long axis of the crown perpendicular to the floor. A metal piston with a tip diameter of 5 mm was applied to the center of the crown perpendicular to the occlusal surface and driven at a crosshead speed of 1 mm/min (Fig. 1). The maximum load to fracture value was recorded by the software automatically in Newton (N).

### CIE L*a*b* parameters and change of color (∆*E*) after aging

In order to investigate the effect of thermomechanical aging on the color, the L*, a*, and b* color parameters of the ceramic crowns were measured under a standard illumination D65 by using a digital spectrophotometer (VITA Easyshade V, VITA Zahnfabrik GmbH, Bad Säckingen, Germany). The CIE L*a*b* parameters represent the coordinates of color in the color space, where L* refers to lightness, a* coordinate represents the green–red range, and b* coordinate represents the blue-yellow range [34]. Six readings per measurement were taken from the middle third of the buccal surface before subjecting the crowns to the chewing cycles and the mean values were recorded as L*_1_, a*_1_, and b*_1_. The color parameters were re-measured again at the end of the chewing cycles under the same testing and lightening conditions and the mean values were recorded as L*_2_, a*_2_, and b*_2_. The color difference (∆*E*) was calculated according to the following equation:

$$\Delta E={\left[{\left({\Delta L}^{*}\right)}^{2}+{\left(\Delta {a}^{*}\right)}^{2}+{\left(\Delta {b}^{*}\right)}^{2}\right]}^{{~}^{1}\!\left/ \!{~}_{2}\right.}$$

where ∆L* = (*L*_1_ – *L*_2_), ∆a* = (*a*_1_ – *a*_2_) and ∆b* = (*b*_1_ – *b*_2_).

### Color testing

#### Specimens’ preparation

For the translucency parameter (TP) and change of color (∆*E*) from the selected shade measurements, fifty rectangular-shaped specimens (12 × 6.5 × 1.5 mm) were fabricated (*n* = 10/group). Specimens of PLD, FLD, and LMS groups were sectioned from CAD/CAM blocks size C14 shade A3 HT using precision cutting saw (Buehler, Lake Bluff, IL, USA) equipped with 0.6 mm wide diamond cut-off-wheel saw with coolant driven at 2500 rpm and a crosshead speed of 0.080 mm/s. The monolithic zirconia specimens (SMZ and UMZ) were cut by the manufacturer from the body layer of the CAD/CAM blank (shade A3), sintered, and delivered in the required dimensions. The thicknesses of all specimens were confirmed by a digital micrometer (Mitutoyo, Illinois, USA). Thereafter, specimens were flattened and polished using silicon carbide abrasive papers (#500, #1200, and #4000 grit, respectively) in a grinding machine (Exakt 400CS, Norderstedt, Germany) under sufficient water-coolant. Then, the specimens of each group were further treated according to the manufacturer’s recommendations as previously explained in the crown preparation section.

#### Translucency parameter

For measurement of the translucency, the ceramic specimens were measured with the VITA Easyshade V spectrophotometer, and CIE L*a*b* parameters were recorded over black and white backgrounds. Three readings were taken for each measurement. The translucency parameter (TP) was calculated by calculating the differences in color parameters over the black and white backgrounds according to the following equation:

$$TP=\sqrt{{\left({L}_{W}^{*}-{L}_{B}^{*}\right)}^{2}+{\left({a}_{W}^{*}-{a}_{B}^{*}\right)}^{2}+{\left({b}_{W}^{*}-{b}_{B}^{*}\right)}^{2}}$$

where “*W*” refers to coordinates over the white background and “*B*” refers to coordinates over the black background.

#### Change of color ∆*E* from shade A3

The VITA Easyshade V spectrophotometer was used to verify the shade of the ceramic specimens. The “Shade verification” mode was selected and A3 was chosen as the control shade. Six readings were recorded for each specimen and the mean was calculated.

### Fracture pattern analysis

Representative samples were selected after fracture load test to examine the origin of fracture. Fracture surfaces were coated with thin gold/platinum layer using a sputter coater (Scancoat six; Edwards High Vacuum, England, UK). Fractured fragments were then examined under scanning electron microscope (SEM, Philips XL 30 CP, Philips, Eindhoven, Netherland) operated at 10 kV. The spot size was adjusted to 5 and the secondary emission was detected.

### X-ray diffraction

Two representative specimens per ceramic were selected from the fractured fragments of the crowns after the fracture load test: one specimen from the fatigued group and one from the non-fatigued. The collected fragments were pulverized and prepared for X-ray diffraction (XRD) analysis. The XRD data were collected using a Guinier diffractometer (G670, HUBER Diffraktionstechnik GmbH & Co. KG, Rimsting, Germany) with a Cu X-ray tube operating at 40 kV and 30 mA. The spectra were recorded using monochromatic radiation (Cu-Kα1 radiation, *λ* = 1.54051 Ǻ) in the 2-theta range from 5 to 85° for the silicate glass–ceramics and from 5 to 100° for the zirconia with a 0.02° step size at every 2-s interval. The data was transferred to “Match” software (Match V.3, Crystal Impact GbR, Bonn, Germany) for identifying the crystals patterns.

### Statistical analysis

The normality of the data was evaluated using the Shapiro–Wilk test. The data for maximum loading force were statistically analyzed by two-way ANOVA followed with multiple comparisons by post hoc Tukey’s test (*α* = 0.05). Student’s *t*-test was used to compare the CIE L*a*b* parameters before and after thermomechanical aging. One-way ANOVA was used to analyze the ∆*E* and TP data followed by Tukey’s post hoc test for pairwise comparisons. Significance level was set to (*α* = 0.05). Statistical analysis was done using Prism software (PrismV.9, GraphPad Software, San Diego, USA).

## Results

### Load to fracture

**Fig. 2:**
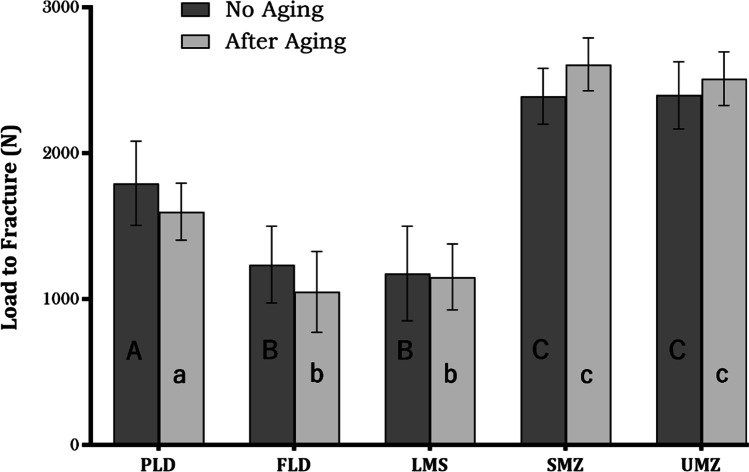
Load to fracture results in newton before and after aging. PLD, partially crystalized lithium disilicate; FLD, fully crystallized lithium disilicate; LMS, lithium metasilicate; SMZ, super-translucent monolithic zirconia; UMZ, ultra-translucent monolithic zirconia. The capital letters show significant difference among groups with no aging. The small letters show the significant difference among groups after aging. Means that do not share the same letter are significantly different. Two-way ANOVA followed by Tukey's multiple comparisons test. Significance level at *p* < 0.05

Only one specimen from FLD group failed (cohesive failure) during the thermomechanical cyclic fatigue aging. Therefore, all the fatigued crowns (except the failed specimen) were subjected to static loading until fracture. According to two-way ANOVA analysis, the material type had a significant effect on the load-bearing capacity of the tested crowns (*p* < 0.001), while the effect of thermomechanical aging was not statistically significant for all groups (*p* = 0.58). For the non-fatigued crowns, the two monolithic zirconia groups (SMZ and UMZ) showed the highest mean fracture load (2390 ± 191 N) and (2379 ± 230 N) respectively, which was statistically significantly higher than all other tested groups. There was no statistically significant difference between LMS and FLD groups (*p* > 0.99) which showed the least mean fracture load (1176 ± 323 N) and (1237 ± 263 N), respectively. The mean fracture load values for PLD (1794 ± 288 N) were lower than those of the zirconia groups and higher than those of the silicate glass–ceramics. The differences between the mean fracture load values of fatigued and non-fatigued crowns were statistically insignificant for all groups (Fig. 2).

### Change of color (∆*E*) after thermomechanical aging

**Fig. 3:**
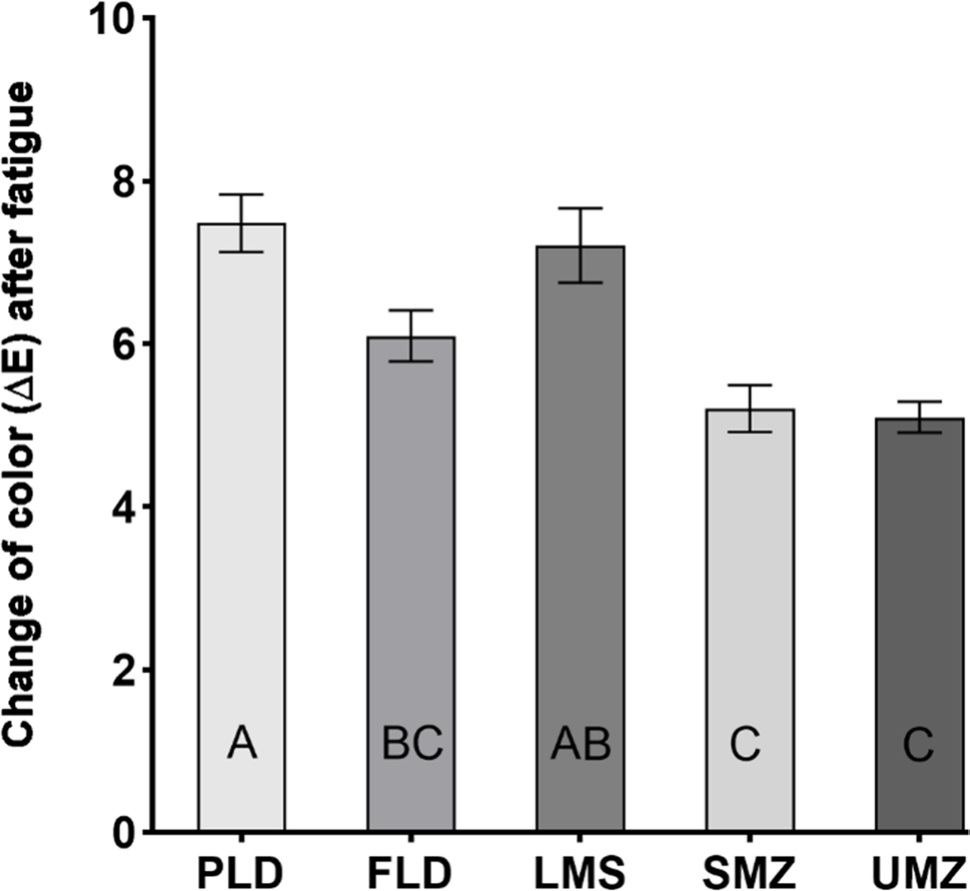
Mean and standard deviations of the ∆*E* for ceramic crowns after thermomechanical aging. PLD, partially crystalized lithium disilicate; FLD, fully crystallized lithium disilicate; LMS, lithium metasilicate; SMZ, super-translucent monolithic zirconia; UMZ, ultra-translucent monolithic zirconia. The letters show the significant difference among groups. Means that do not share the same letter are significantly different. One-way ANOVA followed by Tukey’s multiple comparisons test. Significance level at *p* < 0.05

Means and standard deviations of the L*, a*, and b* values for the ceramic crowns before and after thermomechanical loading are presented in Table 3. The *t*-test for each parameter within each group showed a statistically significant difference after thermomechanical aging in all color parameters for all tested groups (*p* < 0.001). Analysis of variance test showed statistically significant differences between the mean ∆*E* values of the tested crowns (*p* < 0.001). The lowest mean ∆*E* was recorded for the SMZ (5.20 ± 0.91) and UMZ (5.10 ± 0.61). FLD showed a statistically non-significant difference in the mean ∆*E* (6.09 ± 0.94) compared to all other groups except PLD (*p* = 0.04). The difference between SMZ and UMZ groups as well as between PLD and LMS groups was statistically non-significant (Fig. 3).

**Table 3:** Mean and standard deviation of CIElab color parameters of the crowns before (A) and after (B) thermomechanical aging

		PLD	FLD	LMS	SMZ	UMZ
L*	A	70.85 (0.60)	71.47 (1.01)	63.80 (2.10)	75.66 (1.90)	73.37 (0.63)
	B	77.41 (1.19)	75.78 (1.23)	70.08 (1.21)	80.33 (0.77)	77.76 (0.83)
a*	A	− 1.76 (0.05)	− 3.34 (0.16)	− 2.34 (0.13)	− 1.33 (0.46)	− 2.66 (0.22)
	B	− 0.78 (0.11)	− 1.62 (0.24)	− 1.13 (0.09)	− 0.49 (0.46)	− 1.81 (0.22)
b*	A	9.42 (0.13)	15.67 (0.88)	7.59 (0.94)	21.50 (1.12)	15.41 (0.92)
	B	12.85 (0.46)	19.69 (1.01)	11.71 (0.62)	22.32 (1.15)	17.80 (0.89)

L* = lightness, a* = color coordinate that represents the green–red range, and b* = color coordinate that represents the blue-yellow range. A = before aging, B = after aging. *PLD*, partially crystalized lithium disilicate, *FLD*, fully crystallized lithium disilicate; *LMS*, lithium metasilicate; *SMZ*, super-translucent monolithic zirconia; *UMZ*, ultra-translucent monolithic zirconia. *T*-test for each parameter within each group showed a statistically significant difference after thermomechanical aging in all color parameters for all tested groups (*p* < 0.001)

### Fractographic analysis

**Fig. 4:**
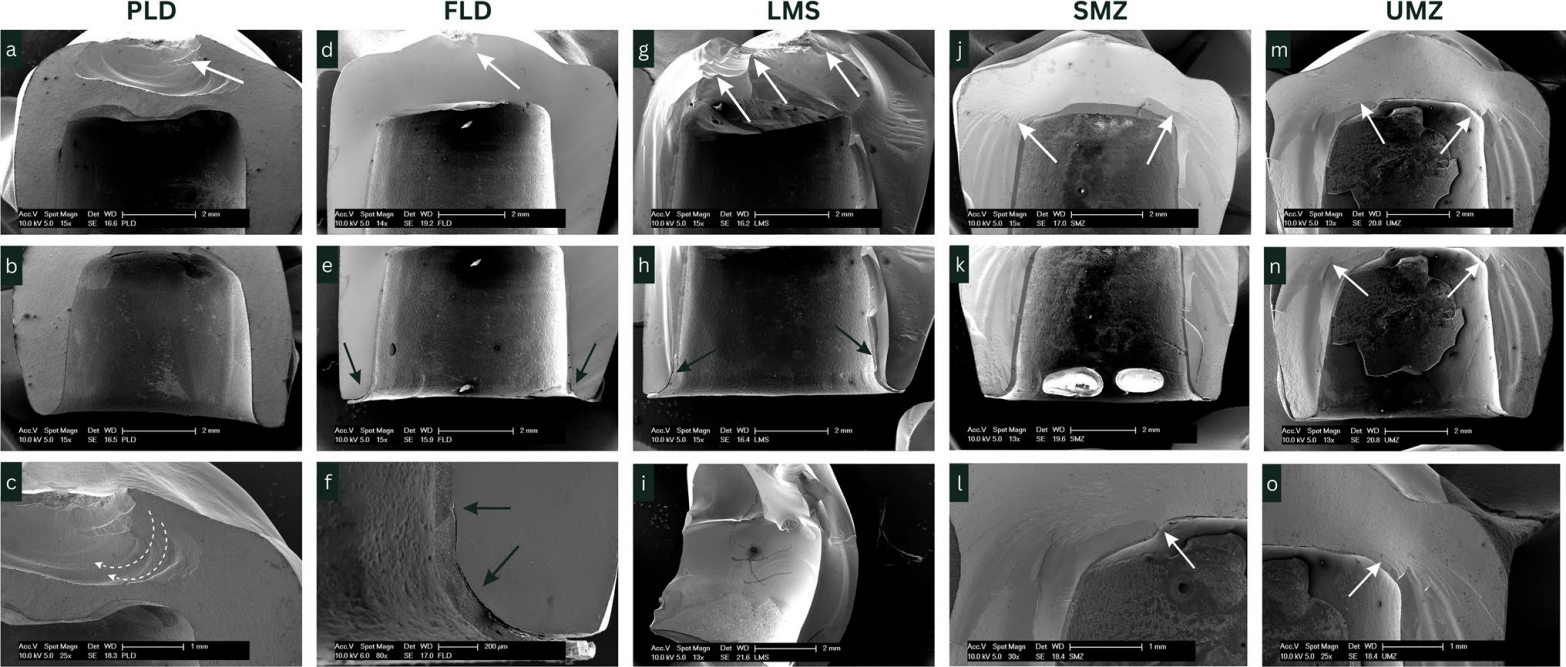
SEM images of selected fractured segments after fracture loading test showing the fracture patterns. PLD, partially crystallized lithium disilicate; FLD, fully crystallized lithium disilicate; LMS, lithium metasilicate; SMZ, super-translucent monolithic zirconia; UMZ, ultra-translucent monolithic zirconia. PLD showed crescent-shaped fracture (dotted arrow). White arrows point to fracture origins starting from tensile regions. FLD and LMS demonstrated cervical fractures 1 mm below the cervical margin (black arrows)

SEM images are presented in Fig. 4. In PLD, cracks were concentrated near the occlusal surface directly below the loading point (Fig. 4a). They showed a form of crescent-shaped fracture lines radiating outward from the loading spot (Fig. 4c). FLD showed similar crack origin, but it was more confined to the load area (Fig. 4d). LMS showed crescent-shaped fracture form below the occlusal surface similar to that shown for PLD (Fig. 4g). Furthermore, it showed multiple cracks extending from the occlusal surface along the axial walls to the cervical area (Fig. 4i). Axial walls showed multiple hackles radiating from the inner surface, namely from tensile zones outward suggesting secondary fractures (Fig. 4h). Both FLD and LMS showed cracks at the cervical margin originating from the inner surface approx. 1 mm below the margin (Fig. 4e, f, h). Fracture patterns of SMZ and UMZ were identical. Both zirconia groups demonstrated cracks originating from the inner occluso-axial line angles and extending along the axial walls (Fig. 4j–o). In summary, it can be stated that the fracture of all-ceramic restorations started on the tensile side and led to the fracture as the crack propagated.

### X-ray diffraction pattern analysis

**Fig. 5:**
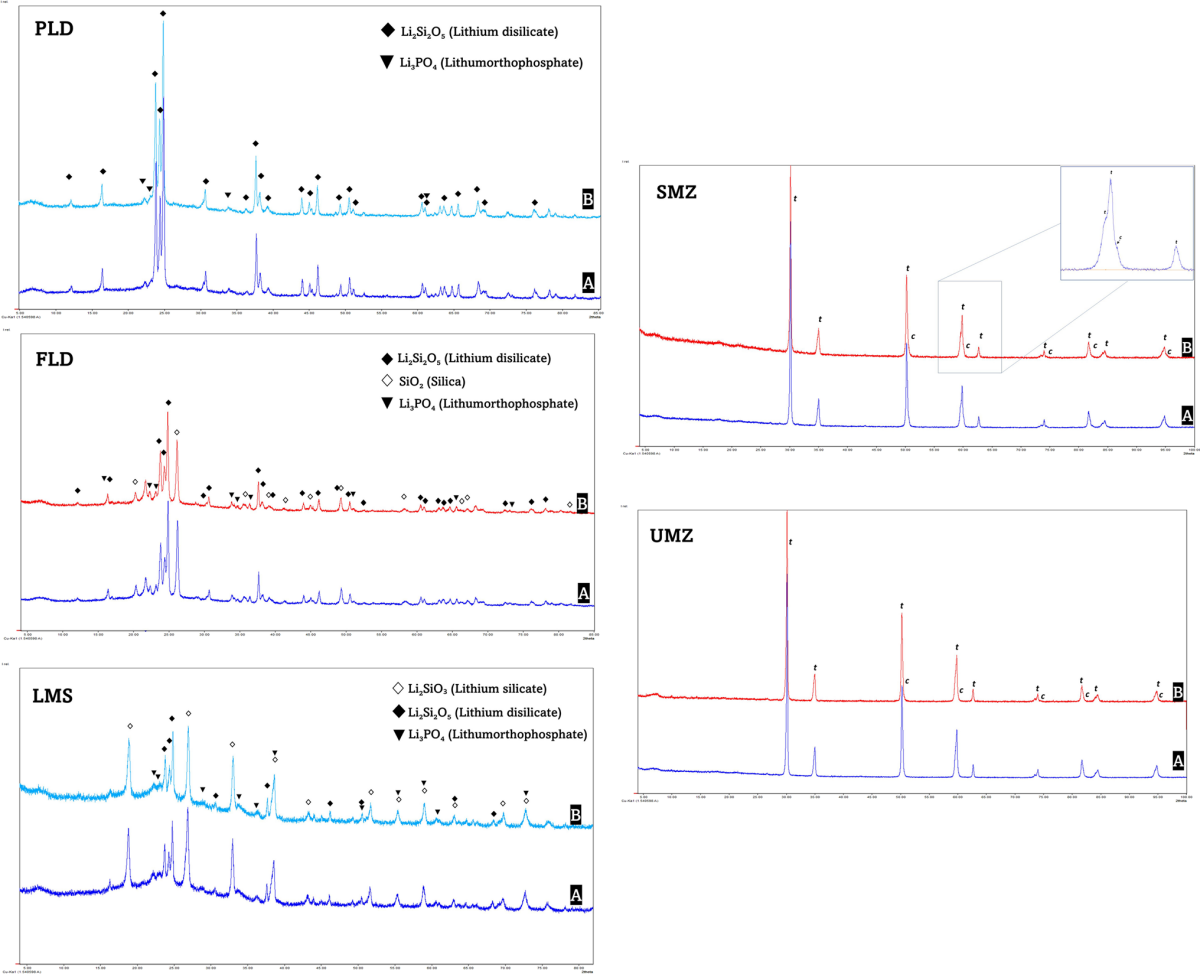
XRD patterns of tested groups. PLD, partially crystalized lithium disilicate; FLD, fully crystallized lithium disilicate; LMS, lithium metasilicate; SMZ, super-translucent monolithic zirconia; UMZ, ultra-translucent monolithic zirconia. A = before aging, B = after aging, t = tetragonal zirconium oxide, c = cubic zirconium oxide

The XRD analysis presented no differences in the crystalline morphology for any of the tested materials after thermomechanical aging (Fig. 5). Lithium disilicate (Li_2_Si_2_O_5_) and lithium orthophosphate (Li_3_PO_4_) crystals were identified in all the silicate glass–ceramic groups to varying degrees. Additionally, lithium silicate (Li_2_SiO_3_) was identified in the LMS pattern, whereas FLD showed the presence of silica crystals (SiO_2_). In terms of zirconia groups, the lattice patterns of the SMZ and UMZ were quite similar. The SMZ specimen showed a lattice pattern that fits very well with the tetragonal zirconia. There was clear splitting in the tetragonal peaks showing additional peaks at 2theta 50.61, 59.82, 74.44, 82.35, and 95.28 which fit with cubic zirconia. The UMZ showed a lattice pattern that matched with the tetragonal phase of yttrium-doped zirconia. There was no clear splitting in any of the tetragonal phase peaks, therefore no additional peaks could be identified.

### Translucency parameter

**Fig. 6:**
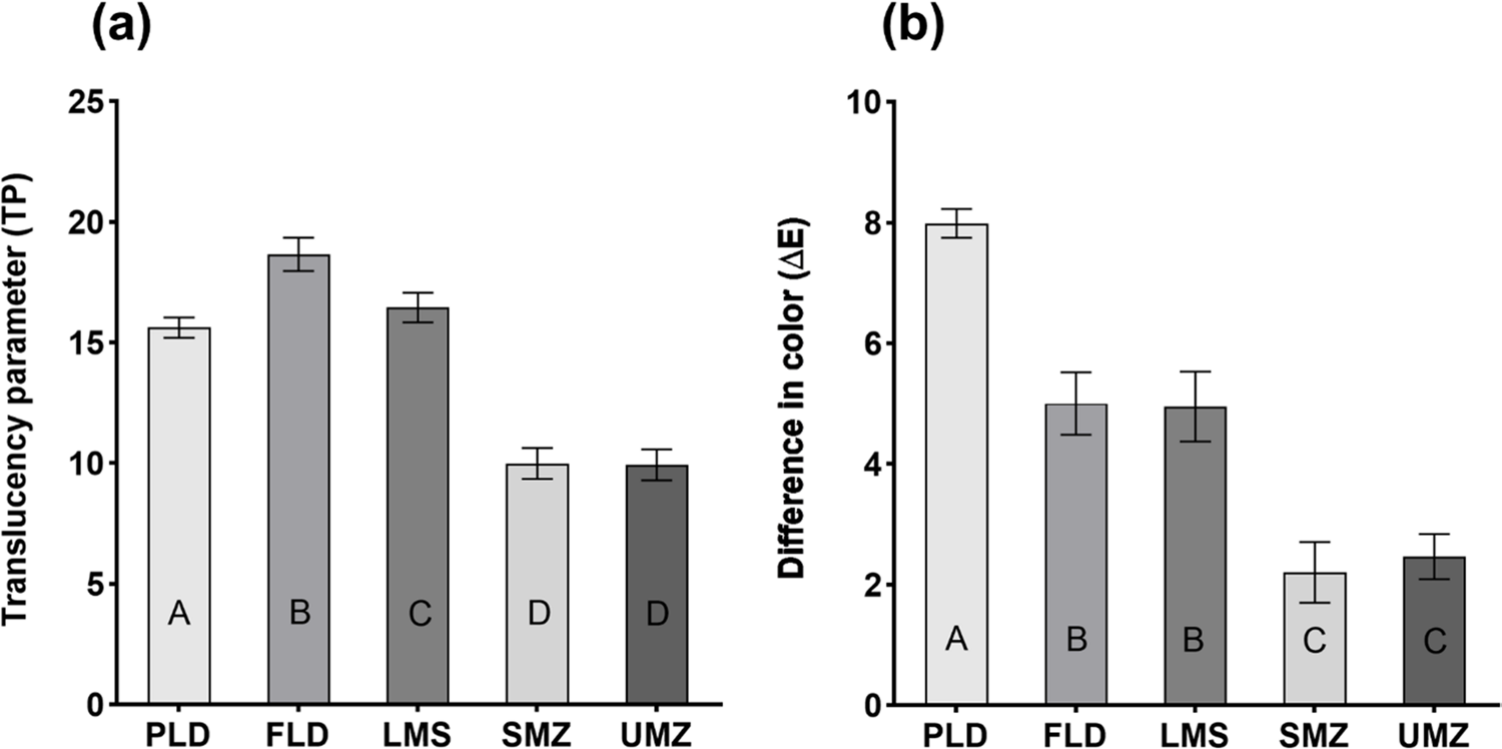
(**a**) Translucency parameter values and (**b**) difference in color (∆*E*) from selected shade A3 of the ceramic specimens. PLD, partially crystalized lithium disilicate; FLD, fully crystallized lithium disilicate; LMS, lithium metasilicate; SMZ, super-translucent monolithic zirconia; UMZ, ultra-translucent monolithic zirconia. The letters show the significant difference among groups. Means that do not share the same letter are significantly different. One-way ANOVA followed by Tukey’s multiple comparisons test. Significance level at *P* < 0.05

One-way ANOVA showed a statistically significant difference between the mean TP values of the tested groups (*p* < 0.001). FLD showed the highest mean TP values (18.7 ± 0.7), while both monolithic zirconia groups SMZ and UMZ reported the least values (9.99 ± 0.6 and 9.93 ± 0.6, respectively). PLD and LMS showed mean TP values (15.6 ± 0.4 and 16.5 ± 0.6), respectively. The differences between all groups were statistically significant (*p* < 0.001) except for the SMZ and UMZ groups which showed non-significant differences (*p* > 0.99). In addition, the difference between the PLD and LMS groups was only marginally significant (*p* = 0.03) (Fig. 6a).

### Difference in color (∆*E*) from selected shade (A3)

Only the zirconia groups showed ∆*E* values below the reported clinically perceptible level (> 3.7). The ∆*E* values between the shade of ceramic specimens and the selected shade (A3) showed statistically significant differences between the tested groups (*p* < 0.001) (Fig. 6b). The highest mean ∆*E* value was reported for the PLD group (7.99 ± 0.24) followed by the FLD group (5.00 ± 0.52) and LMS group (4.99 ± 0.58). The monolithic zirconia groups SMZ and UMZ showed the lowest mean ∆*E* values (2.2 ± 0.5 and 2.47 ± 0.47 respectively). There was no statistically significant difference neither between SMZ and UMZ (*p* = 0.58) nor between FLD and LMS (*p* > 0.99).

## Discussion

The present study showed a statistically significant effect of the material on the fracture load and color stability of the tested crowns. Thermomechanical aging did not significantly affect the maximum fracture load, but it significantly affected the color parameters. There were statistically significant differences in translucencies and (∆*E*) values from selected shade (A3) between the tested materials. Based on these results, the first, third, and fourth null hypotheses were rejected, while the second null hypothesis was accepted.

The failure of restorations under cyclic loading at forces below their ultimate flexural strength has been previously reported [35]. Accordingly, all crowns in the present study were subjected to thermomechanical aging to obtain more realistic predictions of their intraoral performance [36]. A control group was added to identify the aging effect on fracture load and color. The aging method used in the current study is accepted and commonly used in in vitro studies [33, 37–39]. A combination of cyclic pre-loading and thermocycling, simulating the intraoral fatigue process, is considered a clinically relevant approach for testing the durability of dental restorations [36]. In order to allow standardization and to exclude the effect of substrate and cement on the fracture load [40], identical duplicated epoxy dies were used. Furthermore, a static load was applied to the crowns during cementation to ensure uniform cement thickness.

Clinically, the maximum biting force in the posterior region for a normal patient ranges from 490 to 520 N [41], whereas in a patient who suffers from bruxism it can reach up to 790 N [42]. Restorations that can withstand mastication loads of approximately 500 N in the premolar area and 900 N in the molar region could be considered a favorable material for posterior indications [36]. The crowns tested in the current study reported average fracture loads of 1176 to 2397 N without aging and 1051 to 2609 N after aging; therefore, all the materials demonstrated sufficient load to withstand the clinical masticatory forces.

The cyclic loading fatigue did not have any significant effect on the fracture resistance of the tested crowns. After aging, the mean fracture loads of the crowns did not significantly decrease, which indicates that all the tested materials could maintain their mechanical fracture load during function. The X-ray diffraction (XRD) analysis of the tested crowns supported this finding, showing similar patterns for each material regardless of the aging procedure. XRD analysis results of zirconia specimens were similar to the findings of Pereira et al. [43] who reported no alteration in the XRD patterns of Katana STML and Katana UTML after aging.

In contrast to our findings, Garoushi et al. [30] observed a significant reduction in the fracture loads of silicate glass–ceramics after thermomechanical fatigue. In their study, the mean fracture load of PLD decreased from ~ 1280 to ~ 1050 N, FLD from ~ 1400 to ~ 900 N, and LMS from ~ 1300 to ~ 900 N. Different crown designs, cyclic fatigue protocols, and direction of force used in the two studies may be responsible for the differences in results. Their study examined anterior crowns, and loading forces were applied in an inclined direction.

In the present study, monolithic zirconia demonstrated significantly higher mean fracture load values compared to glass-based ceramics which was in accordance with several previous studies [31, 32, 44]. Fractographic analysis showed different fracture patterns for zirconia and glass–ceramics. The origins of fracture in the zirconia crowns were located away from the loading area which indicates a higher ability of the material to resist the falling load. According to SEM images, cracks started from the inner surface at points near the occluso-axial line angles and extended along the axial walls. On the other hand, in glass–ceramic crowns, fractures were related to the loading areas. In the PLD and LMS specimens, the fracture was extended below the surface in a crescent-shape which is characteristic for fatigue fracture [45]. The reason for initiation of cracks at the tensile zones may relate to the adhesion protocols and should be studied further.

Comparing our results to previous studies, Johannsson et al. [32] reported higher mean fracture load values for monolithic zirconia 3038 N ± 264 (Z-CAD, Metoxit) compared to lithium disilicate 1856 N ± 161 (IPS e.max press, Ivoclar Vivadent) after aging. Sun Ting et al. [44] and Nakamura et al. [31] also reported that the fracture resistance of monolithic translucent zirconia crowns exceeds that of lithium disilicate. They reported similar fracture load values for the lithium disilicate 1863 N and 1856 N. However, the zirconia crowns were fractured at loads much higher than ours, recording 4110 N and 5558 N without aging. The differences in zirconia, die material, cement type, and crown design could explain the differences in values. They tested the fracture resistance of mandibular molar crowns, while in our study, premolar crowns were utilized.

According to previous studies that employed XRD analyses, 4­YZP and 5-YZP contain primarily cubic phases [46, 47]. That was not confirmed in the current study as the XRD analysis of the SMZ and UMZ specimens showed mainly tetragonal phases. However, there were clear splitting in the tetragonal peaks of the SMZ pattern at 2theta 50°, 60°, 75°, 82°, and 95° that corresponded to cubic phases with relatively small lattice parameters. Similar findings were reported by Inokoshi et al. [47]. Although no additional peaks or clear splitting were observed in the UMZ, we can anticipate that the resulting cubic phase might have a lattice parameter that is quite similar to the tetragonal phase, perhaps with total superimposition. This assumption supports the findings of Camposilvan et al. [46] who reported that the large tetragonal peaks in the UMZ are actually triplets consisting of a single dominant cubic peak between the two tetragonal doublets, indicative of a large volume fraction of cubic phase.

In the present study, the PLD group had higher fracture load than LMS which confirmed results from a previous study by Choi et al. [48] The microstructure plays an important role in the fracture behavior of ceramic materials [49]. The inclusion of crystalline phases into the glass matrix aimed to improve the mechanical properties of dental ceramics [50]. Furthermore, the type, size, and distribution of crystalline phases define the final characteristics of the material [51]. The densely distributed and highly interlocking needle shape lithium disilicate crystals in PLD improved the fracture resistance through crack deflection [13, 52]. The lower fracture load of LMS compared to PLD could be explained by the lower density of lithium disilicate crystals in the former. XRD patterns showed a higher percentage of the weaker lithium metasilicate crystals in the LMS pattern.

In the current study, FLD crowns showed significantly lower fracture load compared to the PLD group. The FLD crowns were milled from new fully crystallized lithium disilicate CAD/CAM blocks. These new blocks have been advertised as needing only polishing after milling and no subsequent crystallization firing is required [14]. No sufficient data is yet available in the literature about the microstructure of FLD to compare it to the conventional PLD. The difference in fracture load could be attributed to the difference in fabrication methods between the two LDs that might influence the physical and mechanical properties of the final restoration [48]. Moreover, ceramics are brittle materials and thereby susceptible to the initiation of internal cracks and surface flaws during hard machining [53]. Further laboratory studies on the mechanical, physical, and microstructure characteristics of the FLD are recommended.

Only a few studies in the literature focused on the effect of aging on the optical properties of dental ceramics, although it plays a significant role in the long-term success of esthetic restorations [26, 54]. In the current study, thermomechanical aging had a significant effect on the color of tested crowns. The changes in the CIE L*a*b* color parameters demonstrated that the color of all tested crowns became more reddish and yellowish after aging. Statistical analysis indicated a significant change of color (∆*E*) higher than the reported clinically perceptible range (> 3.5) [24] for all tested crowns after simulated aging. Zirconia groups showed more color stability, whereas PLD and LMS crowns reported the highest ∆*E* after aging. A limitation of this study is the measurement of color changes on crowns cemented to epoxy resin dies, where changes in the die and/or cement color could affect final results which needs to be further studied. As silicate glass–ceramics are more translucent than zirconia, the effect of cement and/or die color could be exaggerated.

The color difference (∆*E*) from the selected shade A3 was measured to examine the reproducibility of the desired shade from pre-shaded CAD/CAM blocks. Although the tested CAD/CAM blocks were classified by the manufacturers as A3 shade, they showed different L*a*b* values. The silicate-based glass–ceramic groups showed (∆*E*) values above the clinically perceptible level (> 3.7). The worst shade matching was reported for PLD (∆*E* = 7.99), which was considered clinically unacceptable and could be detected by even untrained observers. On the other hand, zirconia groups were able to produce acceptable matching to the selected shade (∆*E* =  ~ 2.5) without any further characterization step. The fact that different ceramic brands with similar designated shades can produce perceivably different colors has been previously reported [24, 55–57]. Shade matching remains a challenging task for both dentists and technicians. Based on our results, it is apparent that color definition differs relatively among manufacturers, and that color standardization is lacking. Choosing the correct block shade is therefore complicated. If possible, the appropriate shade guide should be used for each ceramic block, and a further characterization step may be inevitable.

In terms of aesthetics, translucency is one of the chief controlling factors and crucial consideration in the selection [24, 58]. Natural-looking all-ceramic restoration must have comparable color and translucency to that of natural teeth [59]. In the present study, silicate glass–ceramic specimens showed higher translucency parameter (TP) values compared to the zirconia ones, which is in agreement with several previous studies [27, 60–62]. The superior translucency of silicate glass–ceramics is attributed to their glass content [7]. Despite the significant improvement in the translucency of new generations of monolithic zirconia over conventional ones, it is still below that of lithium disilicate when measured at the same thickness [27, 63]. Kurt and Turhan Bal [27] reported similar TP values for lithium disilicate (15.63 ± 1.29) and polished monolithic zirconia (8.54 ± 0.49) at thickness 1.5 mm. Similarly, Harada et al. [60] reported higher transmittance of light (Tt%) of lithium disilicate (Emax CAD LT) than all tested monolithic zirconias including SMZ and UMZ at thicknesses 0.5 mm and 1 mm. However, in the same study, the translucency of the SMZ and UMZ zirconia specimens at thickness 0.5 mm was higher than that of lithium disilicate at thickness 1 mm. Church et al. [34] reported similar TP values for high translucency lithium disilicate (17.9 ± 0.2) and different monolithic zirconia ceramics (from 9.2 ± 1 to 11.7 ± 0.7). Since the restoration thickness significantly affects the translucency, the new monolithic zirconia ceramics could be aesthetically comparable to lithium disilicate ceramics when used at their clinically recommended thickness.

In the present study, FLD specimens showed higher TP values than PLD or LMS. This could be related to differences in the type and percentage of crystalline phases. Ceramics with a low crystalline phase are generally considered to have greater translucency [24]. As revealed by XRD, the FLD possessed a smaller percentage of lithium disilicate (Li_2_Si_2_O_5_) crystals than PLD, and silica (SiO_2_) crystals were detected. In addition, the omission of the firing step for FLD might have influenced the translucency [64].

The set-up with the epoxy abutment used could have a negative impact on the fracture load results. It has been previously reported that the fracture loads of restorations decrease on rigidly mounted abutments compared to non-rigidly mounted ones [65, 66]. Also, the elastic modulus of the abutment has an influence on the fracture load of FDPs [65, 66]. Increasing the elastic modulus of the abutments results in increased fracture load of ceramic restorations [67]. Therefore, clinical studies are needed to support these results.

## Conclusion

From this study, it could be concluded that the ceramic materials tested can withstand forces higher than the intraoral masticatory forces. The new high translucent generations of zirconia had higher fracture load than silicate glass–ceramics. The new FLD ceramic is more translucent than PLD, but less fracture resistant. Out of the tested ceramics, only zirconia specimens were able to match the selected shade. There were no standard color coordinates for the color shades of the pre-shaded CAD/CAM ceramic blocks of different manufacturers.

## Supplementary Information

Below is the link to the electronic supplementary material.

Supplementary file1 (PDF 132 kb)

## Data Availability

Data available through contact with corresponding author upon reasonable request.
