# Supplementary figures and images for: An investigation on fatigue, fracture resistance, and color properties of aesthetic CAD/CAM monolithic ceramics
Source: Clin Oral Investig. 2022 Dec 27;27(6):2653–65. doi: 10.1007/s00784-022-04833-y (PMC10264477; doi:10.1007/s00784-022-04833-y)

Suppl. 1 Schematic diagram showing the testing design

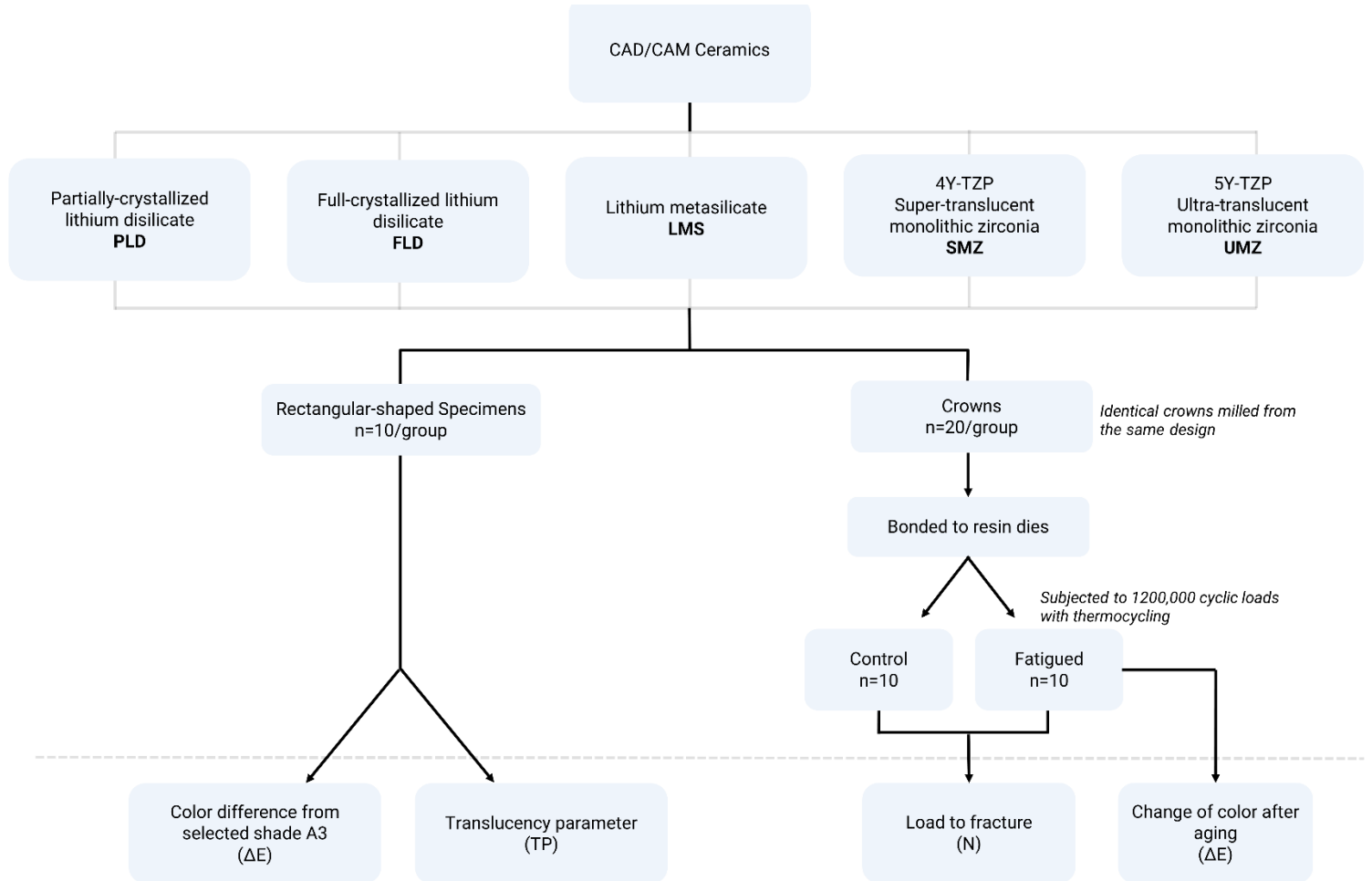

Supplement: Supplementary file 1 — Supplementary file1 (PDF 132 kb) [file 784_2022_4833_MOESM1_ESM.pdf]
